# Draft genome sequences of *Bacillus subtilis* ATCC 6051 strains differing in colony morphology

**DOI:** 10.1128/mra.01361-25

**Published:** 2026-04-02

**Authors:** Gabriel Strong-Lundquist, Eduardo Robleto

**Affiliations:** 1 Department of Life Sciences, University of Nevada, Las Vegas, Nevada, USA; University of Wisconsin-Madison, Madison, Wisconsin, USA

**Keywords:** *Bacillus subtilis*

## Abstract

We report the draft genomes of two morphologically distinct variants of *Bacillus subtilis* (Ehrenberg) Cohn ATCC 6051, an undomesticated reference strain. Despite identical 16S rRNA sequences, the isolates exhibit differences in biofilm-associated genes. Investigating the genomic differences of these colony morphology variants in this model organism can provide valuable insights.

## ANNOUNCEMENT

*Bacillus subtilis* is a gram-positive model organism used to study cell differentiation, stress responses, and biofilm development. Many domesticated laboratory strains are used as models but have accumulated mutations that reduce their environmental fitness (1). Therefore, undomesticated strains, such as *B. subtilis* ATCC 6051 (NCIB 3610), are more appropriate as models when studying ecologically relevant questions.

**Fig 1:**
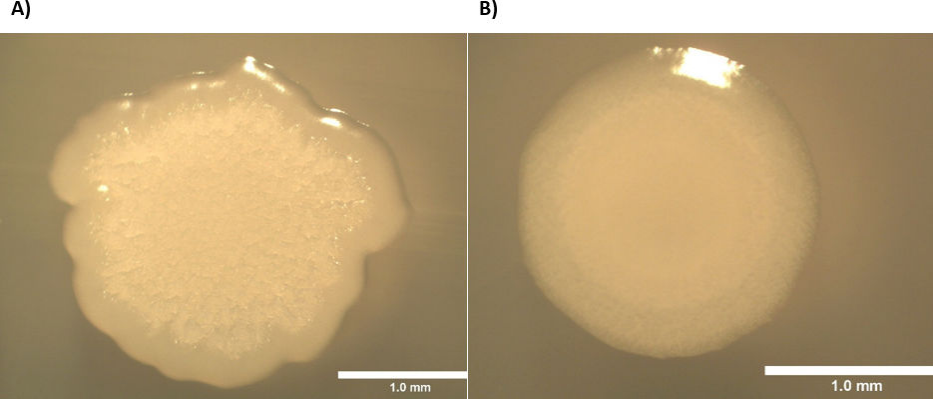
Colony morphologies of (**A**) 6051(R) and (**B**) 6051(S).

Interestingly, two distinct colony morphologies of ATCC 6051 have been observed, namely, 6051(R), a wrinkled, rough-edged form, and 6051(S), a smooth, circular-edged form (Fig. 1), yet both have been confirmed to be *B. subtilis* by 16S rRNA sequencing (2). The morphology variance suggested underlying genomic differences between these variants. To investigate this possibility, we sequenced and annotated the genomes of both 6051(R) and 6051(S).

*B. subtilis* ATCC 6051 was obtained from the American Type Culture Collection (ATCC) and maintained as glycerol stocks at –80°C at the University of Nevada, Las Vegas. Cultures were grown aerobically from single colonies in lysogeny broth at 37°C with shaking (250 rpm) overnight. Genomic DNA was extracted using the Promega Wizard Genomic DNA Purification Kit (Madison, WI). Sequencing and variant analysis were performed with SeqCoast Genomics (Portsmouth, NH). The library preparation was performed using the Illumina DNA Prep Tagmentation Kit (San Diego, CA). Paired-end sequencing (2 × 150 bp) was conducted on the Illumina NextSeq 2000 platform using a 300-cycle flow cell. A 1–2% PhiX spike-in was included to support optimal base calling. Read demultiplexing, trimming, and run analytics were carried out with DRAGEN (v4.2.7); 6051(S) and 6051(R) had 823,799 and 791,532 total reads, respectively. The 6051(S) isolate was sequenced twice to ensure sufficient coverage; reads were merged using Merge Read Libraries (v1.2.2) (3). Raw sequence quality was assessed with FastQC (v0.12.1) (4), and trimming was performed using Trimmomatic (v0.39); 6051(S) and 6051(R) had 815,560 and 783,123 total reads, respectively (5). High-quality reads were assembled into contigs using SPAdes (v3.15.3) (6, 7), and assembly quality was evaluated with QUAST (v4.4) (8, 9). To assess genetic differences, trimmed reads were aligned to the *B. subtilis* reference genome GCF_006088795.1 using Bowtie2 (v2.5.3) (10). Variant calling was conducted using Breseq (v0.38.3) (11) (Table 1). Genome annotation was carried out using the NCBI Prokaryotic Genome Annotation Pipeline (PGAP) (12). Alternative annotation was performed using RASTtk (v1.073) (13–15) along with the assembly (see reference 16). Unless otherwise noted, all tools were run with default parameters. The draft genome of 6051(R) is 4,249,507 bp across 18 contigs (N50: 840,193; 54× coverage), while 6051(S) is 4,248,918 bp across 17 contigs (N50: 616,572; 57× coverage). Both genomes exhibit a GC content of ~43%. RASTtk predicted 4,516 protein-coding genes in 6051(R) and 4,529 in 6051(S).

**TABLE 1:** Variant calling descriptions of the mutations shared with both 6051(R) and 6051(S) compared to the reference NCIB 3610 and the mutations that are different

Variant mutations in both strains compared to the reference	Variant mutation differences	
	6051(S)	6051(R)
*hemAT* (A419V—transition)	*yjaZ-I-appD* (intergenic region—transition)	*fliY* (S224L—transition)
*motA* (L183I—transversion)	*metI* (G67C—transversion)	*pnbA* (P392L—transition)
*remA* (V28A—transition)	*epsC* (A361V—transition)	*sda* (V44V—transversion)
*xynC* (L19L—transition)	*spsL* (G102W—transversion)	*sinI-I-sinR* (75 bp duplication)
*spoIIP* (E201Q—transversion)	EJJ34_RS22485—DHH family phosphoesterase (S344T—transversion)	
*yhbY* (pseudogene—transversion)		
*pdeH* (pseudogene—8 bp duplication)		
*upp* (Y127F—transition)		

Among the observed genetic differences were variants in genes associated with biofilm development, including *epsC* and a 75-bp duplication affecting the *sinI/sinR* regulatory system—well-established determinants of matrix production and colony architecture in *B. subtilis* (17). These differences offer a compelling explanation for the distinct morphologies observed in the ATCC 6051 variants. This work contributes new genomic resources for an ecologically relevant *B. subtilis* strain and highlights natural genetic variability within a widely used model organism.

## Data Availability

The draft genome project data hve been submitted under BioProject accession number PRJNA1289223. Sequence Read Archive (SRA) accession numbers for 6051(S) and 6051(R) are SRR334450845 and SRR34450844, respectively. The genomes of 6051(S) and 6051(R) have been deposited at GenBank under accession numbers JBUZLE000000000 and JBUZLF000000000, and the versions described in this paper are JBUZLE010000000 and JBUZLF010000000, respectively. Kbase generated assembly can be found at: https://kbase.us/n/221851/41/.
